# Women's views on consent, counseling and confidentiality in PMTCT: a mixed-methods study in four African countries

**DOI:** 10.1186/1471-2458-12-26

**Published:** 2012-01-11

**Authors:** Anita Hardon, Eva Vernooij, Grace Bongololo-Mbera, Peter Cherutich, Alice Desclaux, David Kyaddondo, Odette Ky-Zerbo, Melissa Neuman, Rhoda Wanyenze, Carla Obermeyer

**Affiliations:** 1 Amsterdam Institute for Social Science Research, University of Amsterdam, Amsterdam, the Netherlands; 2 Research for Equity and Community Health Trust, Lilongwe, Malawi; 3 National AIDS/STD Control Programme, Ministry of Health, Nairobi, Kenya; 4 Université Paul Cézanne d'Aix-Marseille/Institut de Recherche pour le Développement, Dakar, Sénégal; 5 Makerere University Department of Social Work/Child Health and Development Centre, Kampala, Uganda. Fellow at Wissenschaftskolleg, 2010-11, Berlin; 6 Programme d'Appui au Monde Associatif & Communautaire de Lutte Contre le VIH/SIDA, Ouagadougou, Burkina Faso; 7 Department of Society, Human Development, and Health, Harvard School of Public Health, Boston, USA; 8 Makerere University School of Public Health, Kampala, Uganda; 9 Faculty of Health Sciences, American University of Beirut, Beirut, Lebanon

**Keywords:** PMTCT, Africa, HIV testing, Counseling, Consent, Disclosure

## Abstract

**Background:**

Ambitious UN goals to reduce the mother-to-child transmission of HIV have not been met in much of Sub-Saharan Africa. This paper focuses on the quality of information provision and counseling and disclosure patterns in Burkina Faso, Kenya, Malawi and Uganda to identify how services can be improved to enable better PMTCT outcomes.

**Methods:**

Our mixed-methods study draws on data obtained through: (1) the MATCH (Multi-country African Testing and Counseling for HIV) study's main survey, conducted in 2008-09 among clients (N = 408) and providers at health facilities offering HIV Testing and Counseling (HTC) services; 2) semi-structured interviews with a sub-set of 63 HIV-positive women on their experiences of stigma, disclosure, post-test counseling and access to follow-up psycho-social support; (3) in-depth interviews with key informants and PMTCT healthcare workers; and (4) document study of national PMTCT policies and guidelines. We quantitatively examined differences in the quality of counseling by country and by HIV status using Fisher's exact tests.

**Results:**

The majority of pregnant women attending antenatal care (80-90%) report that they were explained the meaning of the tests, explained how HIV can be transmitted, given advice on prevention, encouraged to refer their partners for testing, and given time to ask questions. Our qualitative findings reveal that some women found testing regimes to be coercive, while disclosure remains highly problematic. 79% of HIV-positive pregnant women reported that they generally keep their status secret; only 37% had disclosed to their husband.

**Conclusion:**

To achieve better PMTCT outcomes, the strategy of testing women in antenatal care (perceived as an exclusively female domain) when they are already pregnant needs to be rethought. When scaling up HIV testing programs, it is particularly important that issues of partner disclosure are taken seriously.

## Background

The 2001 Special Session of the UN General Assembly committed its 189 member states to provide 80% of pregnant women worldwide with access to Prevention of Mother to Child Transmission of HIV (PMTCT) care by 2010. The aim was to reduce the proportion of infected children born to HIV-positive mothers by 20% by 2005, and then by a further 50% by 2010. These ambitious goals have not been met by the majority of countries in Sub-Saharan Africa. When the UN reviewed its progress in 2010, it was estimated that only 53% of pregnant women living with HIV in Sub-Saharan Africa had received antiretroviral drugs to prevent mother-to-child transmission [1]. Such findings led to a renewed UN commitment to ensure that "pregnant women have access to antenatal care, information, counseling and other HIV services" [2]. This paper analyzes qualitative and quantitative reports from PMTCT clients to assess the quality of information provision and counseling for PMTCT in Burkina Faso, Kenya, Malawi and Uganda, and uses these findings to suggest how services can be improved to enable better PMTCT outcomes.

PMTCT was first proposed as a global health policy in the late 1990s [3,4]. Initially, WHO and UNAIDS recommended a three-pillar strategy: to prevent (1) new infections among parents; (2) unwanted pregnancies among HIV-infected women; and (3) transmission from HIV-infected pregnant women and mothers to children [5]. In 2002, a WHO meeting proposed the inclusion of a fourth pillar: to provide care and support to mothers, their infants and their families [5]. These aims were endorsed at the PMTCT High Level Global Partners Forum in Abuja, Nigeria in 2005 [6], by which time global pressure had made antiretroviral treatment available in resource-poor settings.

That PMTCT would not be easy to implement became clear in 2002 when UNICEF conducted pilot studies in 11 countries [7]. In these studies, an average of 30% of women who visited antenatal care (ANC) sites were *not *informed about PMTCT. Of those women who did receive information about PMTCT, 30% did *not *receive an HIV test. Findings from Kenya and Zambia revealed that one-quarter of tested women did not return for their results. Just over half of the women who tested positive never received prophylactic antiretroviral treatment [7]. These studies were conducted before anti-retroviral treatment became widely available--when being diagnosed with HIV was still equivalent to social ostracism and death in most resource-poor settings.

HIV testing practices have changed dramatically since the advent of large-scale antiretroviral treatment (ART) programs, with provider-initiated HIV testing and counseling programs now commencing many more people on treatment [8]. In the early years of PMTCT in Africa, testing was primarily a tool to prevent transmission to children; in the era of ART scale-up, HIV tests have become gateways to treatment for HIV-positive pregnant women. Testing technologies have also changed. When the UNICEF pilot studies were conducted, PMTCT testing facilities would take blood from patients and send it to laboratories for diagnosis; patients had to return for their results, which partly explained the high dropout rates. Nowadays, nearly all health facilities use rapid testing kits requiring only a finger prick; 30 min later the results are known.

The scaling up of testing and treatment has been accompanied by ethical debates on how best to prepare pregnant women for HIV testing. Some argue that pre-test counseling should be comprehensive--to help clients consciously choose for testing, to prepare for a potential positive outcome, and to disclose and commit to prevention should they be HIV-positive [9]. Proponents of comprehensive counseling fear that routine testing approaches can harm HIV-positive women by exposing them to severe stigma and discrimination, for which they are not prepared. Others suggest that as treatment is now available, HIV tests have become ordinary diagnostic tools, though facilities need to ensure that people feel the freedom to opt out [10,11].

Recent studies in Africa report very high rates of consent for HIV testing within PMTCT programs [12-17]. However, attrition in follow-up continues to be a serious problem [14,18-21]. For example, Manzi et al. found that while 95% of pregnant women attending antenatal care in Malawi tested for HIV, only 45% of HIV-positive pregnant women and 34% of babies born to HIV-positive mothers received ARV prophylaxis [14]. Similarly, Coulibaly et al. found that only 36% of 1,829 HIV-positive pregnant women in a PMTCT program in Abidjan, Côte d'Ivoire received AZT [20].

Why are so few HIV-positive pregnant women and their infants receiving ARVs? A qualitative study in Malawi unveiled a variety of institutional and cultural factors behind the low uptake of PMTCT: women unprepared for HIV testing and its implications before visiting the antenatal clinic; fear of stigma, discrimination, household conflict and divorce upon disclosure of HIV status; husbands opposed to testing; long waiting times at antenatal clinics; and high transportation costs for follow-up visits [19]. Painter et al. (2004) found underlying mistrust in health facilities and disbelief in test results to be contributing to the low uptake of prophylactic drugs in an antenatal clinic in Abidjan, Côte d'Ivoire [22]. In Kenya, Delva et al. have pointed to the low quality of PMTCT counseling, with crucial topics such as partner involvement and follow-up support covered haphazardly [23]. Creek et al. observed that post-testing counseling in Botswana evaluated pregnant women for ARV therapy but neglected their psycho-social needs [24].

In this paper, we use qualitative and quantitative data to assess (1) the quality of counseling provided to PMTCT clients, (2) how PMTCT clients perceive their freedom to consent to testing, and (3) the challenges they face in disclosing their status to others. We give an overview of national-level PMTCT policy guidelines, and suggest ways in which care provision can be improved.

## Methods

This paper's findings are derived from the MATCH (Multi-country African Testing and Counseling for HIV) study, the first designed to systematically compare client experiences with HIV testing across countries and modes of testing. Numerous government and donor-supported initiatives have been implemented in Burkina Faso, Kenya, Malawi and Uganda, where HIV testing is increasingly conducted within health facilities. The four countries face similar challenges in expanding their testing regimes: insufficient resources and infrastructure, shortage of adequately trained staff, and difficulties with referral and follow-up. But there are differences in pace of increase (somewhat slower in Burkina Faso) and in the scale of resources invested (larger in Kenya and Uganda).

The MATCH study included a main survey conducted in 2008-09 among clients and providers at health facilities offering HTC services. The study was conducted in the capital region and one rural province or district in each country, where research teams purposefully selected the facilities to include a variety of types of health facilities. Research teams contacted those in charge at each facility, obtained clearance, and asked them to inform clients about the project. Interviews were planned on at least two different days of the week, with an aim of interviewing a total of 15 clients per facility. On the appointed days, interviewers approached clients, described the study, and invited them to participate. The fraction of respondents to be interviewed at each of the selected facilities was based on the expected numbers of respondents per day: at smaller facilities all clients were invited to participate, whereas at busier facilities, every n^th ^client was. The teams had to adjust plans when attendance was low, or when health staff did not present at the clinic. While the full MATCH study includes both women and men, and respondents who have and have not tested for HIV, the current paper reports on the MATCH study's findings as they pertain to the subset of women who tested in an antenatal care facility or because of pregnancy.

The MATCH survey was administered in each country by a team of experienced researchers, who were responsible for training field interviewers and data entry staff, and have provided input into the survey design and interpretation of results. Prior to beginning the interview, respondents were told about the study and asked to provide consent. In most cases, written consent or a thumbprint was given by respondents; however, in a few situations where these methods of obtaining consent were deemed unacceptable by the country team, verbal consent was allowed. Field interviewers were project staff, and were not associated with the health facilities where the interviews were conducted. The interviews were conducted in French (Burkina Faso), English (Kenya, Malawi, Uganda) or local languages. The survey questionnaire included closed and open-ended questions and usually lasted from around 30 min to 1 h. Data were collected on respondents' socio-demographic characteristics and their experiences with HIV testing and counseling, using a checklist of interactions and services during pre- and post-test meetings and during follow-up care. Before entering the data into the database, all questionnaires were checked for consistency.

In this paper we describe the experiences of HIV-positive and HIV-negative pregnant women who were tested for HIV. The full MATCH client sample is comprised of 3,660 respondents. Of these, 2,117 respondents were tested for HIV in or after 2007. Out if this sub-set, 421 respondents were PMTCT testers. Women were categorized as PMTCT testers if they either reported testing in an antenatal care facility or cited pregnancy or PMTCT as their reason for testing in an integrated testing and medical facility, such as a hospital or general health clinic. We excluded 13 PMTCT testers with missing HIV status from the analysis. The final sample therefore included 408 women. These women were generally young (in their 20 s and 30 s) with no or only primary education. In Burkina Faso, educational level of the women was lowest: 40.4% of the respondents had no formal education (see Table 1).

**Table 1:** Age, educational attainment, household assets, marital status, and number of previous HIV tests by country, PMTCT testers

	Burkina Faso		Kenya		Malawi		Uganda		Total	
	**No**.	**Col%**	**No**.	**Col%**	**No**.	**Col%**	**No**.	**Col%**	**No**.	**Col%**

**Age**										

18-25 years	46	44,2	21	28,8	65	47,4	46	50	178	43,8

25-34 years	55	52,9	44	60,3	59	43,1	40	43,5	198	48,8

35-44 years	3	2,9	7	9,6	12	8,8	6	6,5	28	6,9

45 years and more	0	0	1	1,4	1	0,7	0	0	2	0,5

**Education**										

No formal education	42	40,4	0	0	23	16,7	8	8,7	73	17,9

Primary incomplete/complete	36	34,6	31	41,9	92	66,7	43	46,7	202	49,5

Secondary or more	26	25	43	58,1	23	16,7	41	44,6	133	32,6

**Electricity**										

No	68	65,4	24	32,4	131	94,9	46	50	269	65,9

Yes	36	34,6	50	67,6	7	5,1	46	50	139	34,1

**Plumbing**										

No	73	70,2	30	40,5	107	77,5	70	76,1	280	68,6

Yes	31	29,8	44	59,5	31	22,5	22	23,9	128	31,4

**Flush toilet**										

No	102	98,1	42	56,8	132	95,7	87	94,6	363	89

Yes	2	1,9	32	43,2	6	4,3	5	5,4	45	11

**Marital status**										

never married	6	5,8	10	13,5	3	2,2	12	13	31	7,6

married or cohabiting	94	90,4	64	86,5	123	89,1	78	84,8	359	88

divorced/separated	3	2,9	0	0	8	5,8	1	1,1	12	2,9

Widowed	1	1	0	0	4	2,9	1	1,1	6	1,5

**Number of times tested**										

1	57	54,8	35	47,3	66	47,8	34	37	192	47,1

2	32	30,8	26	35,1	43	31,2	24	26,1	125	30,6

3 or more	15	14,4	13	17,6	29	21	34	37	91	22,3

**Total**	104	100	74	100	138	100	92	100	408	100

Two kinds of data were collected from this sub-set of 408 women: multiple choice responses to assess their views and experiences of HIV testing, and answers to open-ended questions which gave women an opportunity to expand on their views and experiences of counseling, consent and confidentiality (the 3Cs).

We used guidelines on HIV testing from major international, United States, and European organizations [25-29] to select variables to measure women's views and experiences of the 3Cs. In light of the debate over the extent of necessary pre-test counseling, and the need to tailor post-test counseling to test results [25,26,30], we examined pre- and post-test counseling separately (see Table 2). In addition, the survey included measures for self-stigma, enacted and community stigma from the WHO's manual *HIV Testing, Treatment and Prevention: Generic Tools for Operational Research *[31], see Table 3. We used Stata 10.1 SE to conduct a descriptive analysis of the 3Cs and measured significance of differences by country and by HIV status using Fisher's exact tests appropriate for the relatively small sample size with a threshold for significance at *p *= 0.05.

**Table 2:** The 3C's variables used in our study

Pre-test counseling
Met with counselor before the test

Provider explained how HIV is transmitted

Provider explained the meaning of HIV-positive and negative results

Given time to ask questions

**Post-test counseling**

Received the results

Provider explained the meaning of the results

Provider gave advice on prevention

Provider suggested to discuss status with someone

Provider advised partner referral

Provider gave time to ask questions

**Consent**

Provider asked if respondent agreed to test

Provider explained that respondent had a choice to accept or refuse

**Confidentiality**

Provider explained that results would not be shared

Respondent believes results have been protected

**Satisfaction with post-test counseling**

Received sufficient information

Was well treated

Thought meeting was useful

**Referral index (HIV positives only)**

Referred for care

Referred to a support group

Prescribed medications

**Table 3:** Stigma variables

Self-stigma
"I sometimes feel worthless because I am HIV-positive"

"I feel guilty because I have HIV"

**Enacted stigma**

"Have you personally ever been made to feel bad because of things people did or say to you on account of your HIV status?"

"Did health workers ignore or avoid caring for you?"

**Reported community stigma**

"Have you heard of people who have been badly treated because they have HIV?"

**Attitudes and behaviors related to disclosure**

"Important that nobody knows results"

"Kept status secret"

We closely examined experiences of stigma, disclosure, post-test counseling and access to follow-up psycho-social support in a sub-set of 63 HIV-positive women (6 in Burkina Faso, 8 in Kenya, 34 in Malawi and 15 in Uganda), using primarily their responses to the open-ended question. And, to gain further insight into these issues, we held 14 in-depth interviews with key informants recruited through support groups for HIV-positive individuals and 21 interviews with healthcare workers in PMTCT facilities. To further probe on the experiences of HIV-positive individuals, the teams conducted 20 focus group discussions with members of support groups (7 in Burkina Faso, 6 in Kenya, 5 in Malawi, and 2 in Uganda). The focus group discussions were facilitated by an experienced qualitative researcher. All interviews except the key informant interviews with HIV-positive women attending support groups and the focus group discussions were held in antenatal care facilities. The interviews and focus group discussions were transcribed and analyzed using NVivo 8 software.

We also reviewed PMTCT policies in the four countries through document study and key informant interviews to determine the guidance given to health workers on how to conduct counseling, and how to confront the challenges of consent, confidentiality and disclosure in pre- and post-test counseling.

The mixed-methods approach of this study allows us to present quantitative measurements as well as qualitative insights on the quality of counseling, consent and confidentiality, and the stigma and disclosure challenges faced by HIV-positive pregnant women. The study was not designed to provide a representative picture of PMTCT implementation in Burkina Faso, Kenya, Malawi and Uganda. It rather aimed to describe policies and practice as experienced by women in antenatal sites and to highlight the issues at stake in further scaling up and increasing access to PMTCT.

The study was reviewed by the ethics committees of the Amsterdam Medical Center of the University of Amsterdam, the World Health Organization, and relevant bodies in each of the four African countries.

## Results

### PMTCT testing policies

The turn towards routine provider-initiated testing has been accompanied by revision of national HIV testing and counseling policies. At the time of our study, all four countries emphasized the principle of informed consent. For example, the 2005 Ugandan policy states that "All HIV testing should be done with the client's knowledge and consent" [32], while the 2008 Kenyan guidelines stress that "No person shall be tested without their consent" [33]. But national policies differ on the role and time allotted to pre-test counseling. The Ugandan policy states that counseling should be "comprehensive" unless the extra time required "causes a barrier to testing itself" [32]. In contrast, the 2008 Kenyan policy places less emphasis on pre-test counseling, implying a form of triage practiced post-test, with those found to be HIV-positive entitled to comprehensive post-test counseling. The Malawian and Burkina Faso policies require pre-test counselors to inform women that they have the *right *to opt out of testing [34,35]. Burkina Faso's national HIV Counseling and Testing (HCT) policy adds that counselors must respect client "autonomy" and understand their reasons for refusing; it recommends improved counseling for more women to accept testing [35].

National policies also differ on confidentiality and disclosure. The 2008 Kenyan HTC policy states that client information cannot be shared without their consent. But the policy--citing evidence that switching from anonymous to confidential HTC does not negatively affect the uptake of HTC services [33]--also states that names rather than codes can now be used in client records to facilitate referral. In contrast, Burkina Faso's policy stresses that test results cannot be shared with third parties, including colleagues, without the client's consent, and that the "principle of autonomy" demands that the client's name be replaced with a code [35]. Uganda's 2005 HCT policy is brief on the issue of confidentiality, stating that "HIV test results and patient records should be kept in a locked file with access limited to HCT personnel" [32]. It further states that the HCT site should not disclose results to anyone without the client's written consent, except when "a court requires it" [32]. Uganda's 2006 PMTCT policy does not mention confidentiality [36]. Malawi's 2008 PMTCT policy also has little to say about confidentiality; it espouses a couple- and family-centered model where health workers encourage shared confidentiality and disclosure among couples [37].

In Kenya and Burkina Faso, legislation--based on a template drafted by parliamentarians from 12 African countries to protect the rights of individuals infected or exposed to HIV [38]--has been passed requiring HIV-positive individuals to disclose to sexual partners. The 2008 Kenyan HTC policy is the most forceful on this point, stating that HTC workers should support their clients to disclose to sexual partners and that "if efforts to encourage the client or patient to disclose their HIV status fail, and the client or patient is placing a sexual partner or other person at risk, a medical practitioner may disclose someone's HIV status to their sexual partner or other person at risk" [33]. Refusal to notify one's partner is thus considered an infringement of the partner's right to health and well-being. Burkina Faso's 2008 HCT policy refers to law N° 030-2008/AN (Article 7), which states that "any person living with HIV is required to promptly disclose their HIV status to their spouse or sexual partner" [35]. But unlike in Kenya, Burkina Faso's policy emphasizes that health workers *cannot *reveal clients' HIV status without their written consent; it encourages health workers to be "patient and understanding with respect to the positive partner who is reluctant to reveal their HIV status to their partner at first" [35].

### Women's views on HIV testing

To explore views on HIV testing, we asked women whether it was hard to be tested. Most (84%) said that it was not. When asked why, their responses reflected prior experience with testing and confidence in a likely HIV-negative result due to their awareness of HIV transmission routes. A 26 year old, HIV-negative married woman from urban Kenya said: "I had been faithful to my husband since testing negative in 2002." It was the same story for a young, HIV-negative married woman from rural Uganda, tested in a government hospital: "Because I had done it before and am also faithful to my husband." A 34 year old, HIV-negative married woman from rural Malawi: "Because I was thoroughly advised to be faithful, which I have been practicing, hence I had no fear."

Some client-respondents said that it was important for the woman to have "made up her mind" before the test. A young educated woman in urban Uganda who refused to be tested stated: "I felt I was not ready for the test that day." As HIV testing is now a common healthcare practice in all four countries, the offering of the test in antenatal care did not come as a surprise to our informants. In fact, 53% of our respondents had been tested more than once (see Table 1). 40% of pregnant women in Malawi, 34% in Kenya, 29% in Uganda and 18% in Burkina Faso had discussed having an HIV test with someone *before *coming to the clinic. In nearly all such cases, they talked to their husbands or partners. In Malawi, some women indicated that their husbands had "encouraged" them to be tested.

The qualitative findings suggest high levels of satisfaction with PMTCT. The services are valued because they help protect the health of the unborn child and because they provide a gateway to treatment. A young HIV-negative woman tested in a government hospital in rural Kenya for example asserted: "It is good... because I was pregnant and wanted to know my status and it is also a must for pregnant mothers." Another young woman tested in a government hospital in rural Uganda added: "It is good because if a mother is found positive she is always given treatment."

Two out of three HIV-positive women said they were glad to know their status and that good things happened to them since learning of their status. They gave various reasons for this. One woman reported that she had learnt to live positively and was able to prevent transmission to her child; another said that her health had improved and that she was still together with her husband. Another woman stated: "I started taking ARVs and I am healthy and nobody can suspect I am positive."

### Experiences with pre and post-test counseling

In all four countries nearly all women (regardless of status) reported that they were treated well and that the PMTCT meeting was helpful overall (see Table 4). The rates for the 3Cs variables (for the total sample) were above 80%, with the exception of two variables: 'time to ask questions post-test' (78% of women were satisfied) and 'advised to discuss their status with others' (69% of women reported that they were advised to disclose).

**Table 4:** Measures of pre-test and post-test counseling, confidentiality, consent, and satisfaction, by country

	Burkina Faso		Kenya		Malawi		Uganda		Total		*P *value*
	**No**.	**Col%**	**No**.	**Col%**	**No**.	**Col%**	**No**.	**Col%**	**No**.	**Col%**	

**Pre-test: meet with counselor before test**											

No	3	2,88	5	6,76	11	8,03	9	9,78	28	6,88	0,220

Yes	101	97,12	69	93,24	126	91,97	83	90,22	379	93,12	

**Pre-test: explain how HIV is transmitted**											

No	8	7,92	4	5,8	5	3,97	6	7,23	23	6,07	0,604

Yes	93	92,08	65	94,2	121	96,03	77	92,77	356	93,93	

**Pre-test: explain the meaning of positive and negative results**											

No	46	45,54	5	7,25	8	6,35	4	4,82	63	16,62	0,000

Yes	55	54,46	64	92,75	118	93,65	79	95,18	316	83,38	

**Pre-test: give time to ask questions in pre-test counseling**											

No	33	32,67	7	10,14	8	6,35	15	18,07	63	16,62	0,000

Yes	68	67,33	62	89,86	118	93,65	68	81,93	316	83,38	

**Found it difficult to test**											

No	79	79	55	79,71	113	91,13	70	84,34	317	84,31	0,044

Yes	21	21	14	20,29	11	8,87	13	15,66	59	15,69	

**Consent: ask if you agreed to test**											

No	6	5,94	4	5,8	10	7,94	11	13,41	31	8,2	0,282

Yes	95	94,06	65	94,2	116	92,06	71	86,59	347	91,8	

**Consent: explain that you had a choice to agree or refuse**											

No	15	14,85	6	8,7	14	11,11	26	31,71	61	16,14	0,000

Yes	86	85,15	63	91,3	112	88,89	56	68,29	317	83,86	

**Confidentiality: explain that results will not be shared**											

No	27	27	8	11,59	4	3,17	4	4,82	43	11,38	

Yes	73	73	61	88,41	122	96,83	79	95,18	335	88,62	0,000

**Confidentiality: believe that results have been protected**											

No	16	15,38	12	16,22	9	6,67	24	26,97	61	15,17	0,001

Yes	88	84,62	62	83,78	126	93,33	65	73,03	341	84,83	

**Post-test: received results**											

No	0	0	0	0	1	0,73	2	2,2	3	0,74	0,348

Yes	104	100	74	100	136	99,27	89	97,8	403	99,26	

**Post-test: information is sufficient**											

No	24	23,08	4	5,41	8	5,88	15	16,85	51	12,66	0,000

Yes	80	76,92	70	94,59	128	94,12	74	83,15	352	87,34	

**Post-test: Explain meaning of result**											

No	3	2,88	5	6,85	2	1,48	4	4,49	14	3,49	0,202

Yes	101	97,12	68	93,15	133	98,52	85	95,51	387	96,51	

**Post-test: give advice on prevention**											

No	32	30,77	9	12,33	5	3,7	7	7,87	53	13,22	0,000

Yes	72	69,23	64	87,67	130	96,3	82	92,13	348	86,78	

**Post-test: advise to discuss status**											

No	76	73,08	11	15,07	21	15,56	18	20,22	126	31,42	0,000

Yes	28	26,92	62	84,93	114	84,44	71	79,78	275	68,58	

**Post-test: advise partner referral**											

No	28	26,92	11	15,07	8	5,93	7	7,87	54	13,47	0,000

Yes	76	73,08	62	84,93	127	94,07	82	92,13	347	86,53	

**Post-test: time to ask questions**											

No	47	45,19	7	9,59	14	10,37	19	21,35	87	21,7	0,000

Yes	57	54,81	66	90,41	121	89,63	70	78,65	314	78,3	

**Satisfaction: post-test meeting was helpful overall**											

No	1	0,96	3	4,05	3	2,21	7	7,87	14	3,47	0,054

Yes	103	99,04	71	95,95	133	97,79	82	92,13	389	96,53	

**Satisfaction: treated well overall**											

No	0	0	6	8,11	3	2,21	26	29,55	35	8,73	0,000

Yes	103	100	68	91,89	133	97,79	62	70,45	366	91,27	

*Fischer exact test

The statistical analysis reveals that there are no significant differences between the countries for the variables 'met with a counselor', 'explained how HIV is transmitted', 'counselor asked if you agreed to be tested', 'received results' and 'explained meaning of results'. However, statistically significant differences between the countries were observed for 'pre-test: explained the meaning of HIV positive or negative results', 'time to ask questions', 'explained that results will not be shared', and for advice on prevention, partner referral and disclosure. In Burkina Faso, scores appear to be particularly low on these 'comprehensiveness' variables: only 55% of respondents reported that during pre-test counseling the meaning of results was explained to them; only 67% said they were given time to ask questions pre-test; during post-test even fewer women were given a chance to ask questions (55%); only 69% were given advice on prevention and only 27% of respondents were advised to discuss their status with others. Burkina Faso also has the lowest number of respondents who report that they are satisfied with the information received in PMTCT (77% versus 95% in Kenya and 94% Uganda and 83% in Malawi). A high proportion of our respondents in Burkina have a low educational status (see Table 1), which could explain the lower reported quality of counseling in the country. Perhaps the health workers explain less because they do not expect that their clients will understand the information provided.

We expected that the HIV-positive women would evaluate the services more negatively: they require comprehensive counseling and may have many questions, while health workers are generally pressed for time. But this was not the case. Significantly higher percentages of HIV-positive women reported being given time to ask questions, advised to discuss their status and advised to refer their partners. (see Table 5). Most of the HIV positive women were prescribed HIV medication (85%), and around two-thirds were referred to a support group (see Table 6).

**Table 5:** Measures of post-test counseling, by HIV status

	HIV-negative		HIV-positive		Total		*P*-value*
	**N**	**%**	**N**	**%**	**N**	**%**	

**Post-test: received results**							

No	2	0.6	1	1.6	3	0.7	0.403

Yes	340	99.4	63	98.4	403	99.3	

**Post-test: meaning of result explained**							

No	13	3.8	2	3.2	15	3.7	1.000

Yes	326	96.2	61	96.8	387	96.3	

**Post-test: given advice on prevention**							

No	50	14.7	4	6.3	54	13.4	0.105

Yes	289	85.3	59	93.7	348	86.6	

Post-test: advised to discuss status***							

No	116	34.2	11	17.5	127	31.6	0.008

Yes	223	65.8	52	82.5	275	68.4	

Post-test: advised to refer partner***							

No	51	15	3	4.8	54	13.5	0.027

Yes	288	85	59	95.2	347	86.5	

Post-test: time to ask questions***							

No	84	24.8	4	6.3	88	21.9	0.001

Yes	255	75.2	59	93.7	314	78.1	

**Satisfaction: post-test information was sufficient**							

No	44	12.9	7	11.1	51	12.7	0.837

Yes	296	87.1	56	88.9	352	87.3	

**Satisfaction: post-test meeting was helpful overall**							

No	13	3.8	1	1.6	14	3.5	0.706

Yes	327	96.2	62	98.4	389	96.5	

**Satisfaction: treated well overall**							

No	30	8.9	5	7.9	35	8.7	1.000

Yes	308	91.1	58	92.1	366	91.3	

*two-sided Fisher's exact

**Denominator includes only respondents who indicated someone should be told

***significant difference between HIV-positive and HIV-negative respondents (*p *< 0.05)

**Table 6:** Referral to care (HIV-positive PMTCT testers only)

	HIV-positive	
	**No**.	**Col %**

**Referred for care**		

No	19	31,1

Yes	42	68,9

**Prescribed medication for HIV**		

No	9	14,8

Yes	52	85,2

**Referred to support group in post-test counseling**		

No	23	37,7

Yes	38	62,3

### A closer look at experiences with consent

Does the scaling up of testing come at the expense of women knowing that they can opt out, as has been suggested by some earlier studies [39]? In our study, 92% of PMTCT testers reported being asked if they agreed to testing; 84% were informed that they had the right to refuse. But women's experiences varied. A focus group discussion among women who had visited the Comprehensive Care Centre at a National Hospital in Kenya revealed that they could, at least in theory, refuse the HIV test: "You have a choice because you are not being forced." "If you do not want to be tested you can refuse." "There is no pressure to agree to be tested." But one respondent added: "The doctor is very powerful. If he tells you... you have to agree. If a doctor or any other medical person tells you to get tested, you do not have any other alternative but to get tested."

Other women reported that testing was mandatory. A married, 23 year old HIV-negative woman from rural Kenya, tested in a government Health Center, reported: "The nurse said it was mandatory when pregnant for PMTCT." A married, HIV-negative woman from urban Malawi, tested in a government hospital, likewise reported: "We were told that HIV testing and counseling is a must for pregnant women these days: no HTC, no assistance from the doctor." It was the same for a young, uneducated, HIV-negative woman from urban Uganda, tested in a government hospital: "It was mandatory and a prerequisite for me to get treatment." In Burkina Faso, some women who refused the test were asked to sign a form, which was experienced as intimidating.

Many of our respondents did not oppose mandatory testing. When probed on who should be tested, pregnant women were most commonly mentioned, while other answers included unfaithful husbands and sex workers. But other respondents stressed that forcing women to test for HIV was not good. They reasoned that some individuals would not be able to handle the results that women should be able to choose for themselves, and that mandatory testing would scare women away from antenatal care. One rural uneducated woman in Malawi referred to a violation of rights: "It's not good since if she is not ready, her rights can be violated."

Opting out by pregnant women burdens health workers as they then have to keep track of the women and keep offering tests. As a matron in charge of a maternity in Kenya explained:

*There are situations when antenatal mothers come and we are supposed to capture them for testing in their first visit, and when you give the information and counsel them especially on matters related to HIV, some will say, "look, sister, because I did not come with my husband, I would prefer to take the test when we are together." You just release her but on a subsequent visit she will repeat the same story about the husband not consenting, and she won't tell you she is declining. On another visit she lands on someone else's hands and not you who had counseled her, and the same story is repeated, not knowing that one had been counseled earlier and she is refusing indirectly. Finally, you realize that the mother is approaching delivery and has not been tested, like yesterday we had one, she kept on refusing to take the test, one of the counselors called me and narrated the story, we went to the mother and told her to sign that she has refused to take the test.... When we meet such clients we do continue counseling, we never get tired until the client agrees or disagrees..*.

Some health workers revealed that they deal with the challenge of "capturing" women by telling them that the test is mandatory: "They accept once you explain to them that the test is mandatory at ANC", explained an ANC counselor in a Kenyan health centre. An ANC nurse in a Ugandan health center likewise stated: "We talk to them about HIV testing, we tell them about the benefits, we then tell them that it is a government policy for all pregnant mothers to test."

### Stigma and confidentiality

Most HIV-positive women (79%) reported that they generally kept their HIV status secret. When probed about stigma, one out of five respondents reported that they felt worthless due to being HIV-positive; an equal proportion felt guilty. One out of four had been personally made to feel bad, and around half had heard about other HIV-positive people being treated badly. Only one respondent reported that health workers avoided caring for her (see Table 7).

**Table 7:** Measures of stigma and disclosure (HIV-positive PMTCT testers only)

	N	%
**Disclosure**		

**Important that nobody knows results**		

Very important	53	85.5

Somewhat important	4	6.5

Not important	5	8.1

**Kept status secret**		

No	13	20.6

Yes	50	79.4

**Told someone results**		

No	11	17.5

Yes	52	82.5

Told partner results*		

No	32	62.7

Yes	19	37.3

Told family results*		

No	19	37.3

Yes	32	62.7

Told friend results*		

No	46	90.2

Yes	5	9.8

**Stigma and discrimination**		

**Self-Stigma:**		

*feel worthless because HIV+*		

Undecided/disagree/strongly disagree	48	80

Strongly agree/agree	12	20

*feel guilty because HIV***+**		

Undecided/disagree/strongly disagree	47	81

Strongly agree/agree	11	19

**Enacted stigma/discrimination:**		

*personally been made to feel bad*		

No	44	74.6

Yes	15	25.4

*health workers ignored or avoided caring for you*		

*No*	59	98.3

Yes	1	1.7

**Reported community stigma:**		

*heard of people who have been badly treated*		

No	31	51.7

Yes	29	48.3

**Good things happened because status known**		

No	20	34.5

Yes	38	65.5

*Denominator is persons who've told someone their status

The large majority (85%) felt that the health workers and counselors respected their desire for confidentiality by protecting their results (See Table 4). An example of breached confidentiality was given by a 22 year old woman from Malawi, who tested positive in a rural health center when she was pregnant in 2008. A counselor from a support group had disclosed her status to others in the community without her consent: "The counselor of publicized at my village that I've been found positive, which has left many people to know though I took it as confidential."

### Low levels of partner disclosure

In our study, 83% of the HIV-positive pregnant women had disclosed to someone. Mostly they disclosed to family members (63%), usually their sister and/or parents. Only one in three had disclosed to their partners, while one in ten had disclosed to a friend.

Given post-test counseling's emphasis on disclosing to partners, it was surprising that only one-third of the HIV-positive pregnant women had done so. Analysis of the open-ended questions revealed that some of these women were divorced or widowed. But many others reported that they found it very difficult to tell their husbands, out of fear of being blamed, abused and/or abandoned. A 22 year old woman, tested in an urban Kenyan health center and encouraged by health workers to disclose to her husband and convince him to test, stated: "My spouse denied that I was positive and did not take the test. He thought then and still thinks it's a joke." A 29 year old pregnant woman, tested in a government hospital in rural Kenya who has known her HIV-positive status for 16 months said she was afraid to disclose because she expected her husband to beat her up and leave her. It was a similar story for a 19 year old pregnant woman in Uganda; she had learnt her HIV-positive status 3 months prior to our interview and had not disclosed to anybody, including her partner: "my husband would divorce me even if he knows that he is infected." Only a few of the women that we interviewed in Uganda and Kenya were a member of a support group at the time of our interview. A 25 year old HIV-positive woman was encouraged by health workers at an urban health center in Burkina Faso to disclose to her parents. She disclosed to her parents as well as to her brother and husband. Disclosing to her husband did not go smoothly: "I have had to fight with my husband, he told me he had someone else, he would chase me away because I have AIDS and he doesn't. He also told his friends and other relatives that I have AIDS." She joined a support group, as did two of the other six HIV-positive mothers in Burkina Faso.

We interviewed 34 women who tested HIV-positive in Malawi, more than in any other country. Relatively many (13 out of 34) had joined a support group. Eight of them reported having disclosed to their partners. One 30 year old woman described how her husband "chased me out of our house for a week" when she disclosed to him. She is not intending to disclose to anyone else because she doesn't want to be laughed at. A 35 year old woman reported: "my husband left me for another wife." Another woman was divorced after she disclosed. Staff at a rural health center had suggested that she should disclose to her mother and relatives; she feared that they wouldn't accept the results and decided to disclose to her husband instead. He accused her, demanded to know how she got infected, and subsequently left her. But in some cases, the husband grew more accepting over time. One young woman reported that her husband was at first angry and accused her, but now treats her better. A 40 year old woman, tested in an urban government hospital, explained that she disclosed because "it was good to let him know." He is supportive now but gets worried when she falls ill with, for example, malaria. She will not disclose to others because "people might start gossiping whenever they know my status, as a result I will lack peace of mind."

The in-depth interviews revealed that disclosure is not always direct. Some women disclosed by encouraging their partners to go with them to couple's counseling and testing; others disclosed by *not *hiding their antiretroviral drugs.

## Discussion

This four-country study reveals that pregnant women are generally satisfied with HTC in antenatal care. It is valued because it helps protect the health of the unborn child and because it provides a gateway to treatment. Women generally reported that they did not find the HIV test hard to take. Around half of our respondents were repeat-testers and knew what to expect.

The results of our quantitative analysis reveal that overall the majority of pregnant women attending antenatal care (80-90%) report that they were explained the meaning of the tests, explained how HIV can be transmitted, given advice on prevention, encouraged to refer their partners for testing, and given time to ask questions. In all four countries, nearly all women (regardless of their HIV status) reported that they were treated well and that the meeting was helpful overall.

The policy analysis shows that counselors in all countries are expected to ask for consent before conducting an HIV test, but that there are differences in HTC policy regarding the comprehensiveness of pre- and post-test counseling and the requirements of confidentiality. The statistical analysis reveals that there are no significant differences between the countries for the 3Cs variables 'met with a counselor', 'explained how HIV is transmitted', 'counselor asked if you agreed to be tested', 'received results' and 'post test: explained meaning of results', reflecting convergence in policy between the countries on these 'minimal' elements of HTC. However statistically significant differences between countries were observed for other 3Cs variables, including 'pre-test: explained meaning of results', 'time to ask questions' and advice on prevention, disclosure and partner referral. These results reveal that the 'comprehensiveness' of the HTC in ante-natal care services varies between the countries (and probably also by facility within the countries, but because of sample size limitations we could not test for this). Our findings suggest that the quality of counseling is lowest in Burkina Faso, where fully 46% of women said that they were not explained the meaning of HIV positive and HIV negative test results in pre-test counseling, while post-test 45% were not given a chance to ask questions, 31% were not given advice on prevention, and 73% of respondents were not advised to discuss their status with others. These findings may reflect relatively low educational levels among respondents in Burkina Faso and health workers assuming that uneducated women will not understand the information they provide. An earlier study on the quality of HIV testing and counseling care in 14 Burkina Faso health facilities [40] reported remarkably similar findings: 46% of clients were not explained the significance of positive or negative test results in pre-test counseling, and only 44% of clients were not given a chance to ask questions. The limited attention to disclosure in the counseling sessions in Burkina Faso could be related to the emphasis in the Burkina Faso policy on confidentiality. The Burkina Faso HCT policy states that health workers need to be patient and understanding when an HIV positive person does not want to disclose.

While our quantitative assessment of consent showed that pregnant women in all four countries have the option to refuse the test, the qualitative findings suggest that some women feel it is an "offer they cannot refuse." Different views prevail on mandatory testing for HIV. In Uganda and Kenya, we found some providers--and some women--supporting it for pregnant women; underlying their stance is the moral imperative to protect the unborn child. Many women in Malawi and Burkina Faso, in line with these countries' policies, asserted that women should have the right to refuse HIV testing.

We found that healthcare workers in the four countries provided post-test counseling of a significantly higher standard to HIV-positive women than to their HIV-negative counterparts. Even so, 20% of the HIV-positive women in our study reported that they felt guilty or worthless, suggesting that self-stigma continues despite increased access to anti-retroviral treatment in all four countries.

Compared to other studies [41,42] we found relatively high levels of disclosure: 83% of our HIV-positive respondents had disclosed to someone, usually a family member. But disclosure was selective. Women simultaneously told us that they generally kept their status secret. Despite the availability of anti-retroviral treatment, they continue to fear stigma and social suffering if word about their HIV status spreads in the community. As Bond [43] points out, women appear to juggle the pragmatic advantages of disclosing to a limited circle of people with the importance of maintaining their moral integrity. Their underlying fear is to be blamed for being promiscuous, or for introducing the virus into the family. A recent cross-sectional study in rural Kenya [44] found that 37% of pregnant women accepting an HIV test feared that they would be rejected and/or physically abused by their partners if they tested positive and their partners found out.

Only 37% of the HIV-positive women in our study had disclosed to their partners, despite the emphasis on partner disclosure in post-test counseling in all four countries. Analysis of the qualitative data revealed that disclosure can create serious rifts with partners; some women were abandoned or divorced by their husbands. These findings confirm those from earlier studies on the influence of anticipated rejection and violence from boyfriends and husbands on partner disclosure [45-50].

The risk of intimate partner violence is a crucial issue that needs to be addressed in PMTCT programs. Our findings suggest that *enacted *stigma is still prominent among HIV-positive pregnant women in Sub-Saharan Africa, despite the widespread availability of ART. Roura et al. [49] point out that this is due to ART availability only marginally affecting the discourse of blame that lies behind enacted stigma (the authors argue that self-stigma has declined). Women who test positive in PMTCT generally find out when they are not yet ill--earlier than their male sexual partners who tend to be tested when a health worker suggests a diagnostic HIV test. Many HIV-positive pregnant women thus fear being blamed for introducing the infection into the family. A study in rural Malawi found that some women waited until their husband showed signs of illness before disclosing their positive status [51].

While our survey found healthcare workers to be systematically advising pregnant women to refer their husbands for testing, the in-depth interviews revealed that partners rarely come in for testing. This may be due to men's reluctance to be seen in antenatal clinics, which are seen as part of a 'female' domain; attending one would risk questions from peers about one's masculinity [52]. In countries such as Burkina Faso and Kenya where HIV transmission is criminalized, HIV-positive pregnant women could theoretically even be prosecuted for infecting their husbands and children. The potential adverse effects of disclosure for women, particularly during the socially vulnerable period of pregnancy, suggest a need for accompanying measures that promote early testing among men. This is further supported by the findings that men tend to more often disclose to their partners than women [53], and that knowing one's partner's status facilitates disclosure [54].

Now that HIV testing is being scaled up in Burkina Faso, Kenya, Malawi and Uganda, better integration of PMTCT with other modes of testing, such as home-based or workplace HTC, should be possible. The Ugandan MATCH country study found home-based testing to facilitate disclosure [55-58]. If accompanied by good follow-up medical care, home-based testing enables early treatment for both men and women, allowing them to plan for PMTCT *before *pregnancy.

The current study contributes to the development of methods to measure consent, counseling and confidentiality procedures (the 3 C's) in HIV testing. While such studies are common in family planning [59-61], in HIV testing the emphasis has been on measuring the acceptability and uptake of follow-up medical care, with relatively little attention to the interpersonal processes involved.

One limitation of the study is its purposive sampling frame. Our sample was made up of purposively selected antenatal care facilities in the capital city and one rural province/district in all four countries. We aimed to include a range of facilities in the sample to explore diversity in HTC practice. A systematic sample of respondents was taken in each facility, but adjustments in the sampling procedures were made when attendance was lower than expected. The number of ante-natal care clients interviewed per facility varied between 5 and 30. The samples are thus not representative of the population of ante-natal care clients in the countries concerned. Because of low numbers of PMTCT respondents per facility, we could not conduct multi-level analysis aimed at analyzing the effects of country level policies, institutional practices and individual factors on client satisfaction with PMTCT services. Moreover, the data were collected in 2008/2009. While this is more recent than most published studies, the findings do not necessarily reflect the current situation in the countries concerned.

Another limitation of the study is that the interviews were conducted within health facilities. It is possible that the relatively high levels of satisfaction reported in our study were caused by desirability bias; several other studies have shown that clients are reluctant to express negative opinions of services when interviewed at health facilities [62,63]. By adding open-ended questions to the interview and by conducting additional key informant interviews in communities, we were able to triangulate the findings and overcome this limitation. For example, the quantitative data suggested high levels of disclosure, while the answers of HIV-positive pregnant women to the open-ended questions revealed that disclosure is selective and that secrecy remains the norm for fear of stigma and social suffering.

Finally, the relatively small number of HIV-positive individuals included in the study (N = 63) and their uneven distribution (half are from Malawi) does not allow for quantitative analysis of the differences in experience of HIV-positives by country. We have therefore highlighted our qualitative findings for this sub-population, pointing to the common concerns of HIV-positive pregnant women across the diverse institutional and country settings.

## Conclusion

Analysis using mixed methods is important to provide not only measurements of perceived quality, but also insight into women's experiences of these services, as well as the barriers that remain in efforts to stop transmission of HIV from mother to child. Our study reveals that women are generally satisfied with PMTCT services, but that there is a lack of clarity surrounding the necessity and meaning of consent. In Burkina Faso, the quality of counseling was found to be sub-optimal. More in-depth studies are needed to further elucidate the dynamics within PMTCT to understand why, in some cases, women are not provided with good quality care.

The study further found that the majority (63%) of HIV-positive pregnant women do not disclose their status to partners. Some women, who did disclose, faced rejection by their partners and/or divorce. Moreover, 20% of women experienced self-stigma, while 25% reported that they were personally made to feel bad because of their HIV status. These findings suggest that the PMTCT strategy of testing pregnant women in antenatal care--and subsequently asking them to refer their partners--needs to be rethought. Promoting testing options that reach men might reduce (self-)blame among women for introducing the disease into their families and promote disclosure. If accompanied by good follow-up medical care, home-based testing enables early treatment for both men and women, allowing them to plan for PMTCT *before *pregnancy.

## Competing interests

The authors declare that they have no competing interests.

## Authors' contributions

Concept protocol: This analysis is part of a larger project, for which the research proposal was developed by CO. AH designed the analysis of a subset of data on which this paper is based.

Data collection: CO and AH jointly developed the quantitative and qualitative research instruments in consultation with researchers (GB, PC, AD, DK, OK and RW) from the country teams. Data collection was done by the country researchers (GB, PC, DK, OK and RW) together with research assistants.

Data analysis: MN conducted the quantitative analysis with input from AH; EV and AH conducted the qualitative analysis. AH and EV were responsible for the triangulation of results, in which some of the quantitative findings were nuanced with qualitative insights. The conclusions were validated with the country researchers (GB, PC, DK, OK and RW).

Manuscript draft: The manuscript was written by AH with substantial contributions by EV. All authors commented on the first draft of the paper. Comments were incorporated in the final draft, which was sent around for approval before submitting.

## Pre-publication history

The pre-publication history for this paper can be accessed here:

http://www.biomedcentral.com/1471-2458/12/26/prepub
